# Morphological, physiological, biochemical, and transcriptome studies reveal the importance of transporters and stress signaling pathways during salinity stress in *Prunus*

**DOI:** 10.1038/s41598-022-05202-1

**Published:** 2022-01-24

**Authors:** Biswa R. Acharya, Devinder Sandhu, Christian Dueñas, Marco Dueñas, Manju Pudussery, Amita Kaundal, Jorge F. S. Ferreira, Donald L. Suarez, Todd H. Skaggs

**Affiliations:** 1 USDA-ARS, U.S. Salinity Lab, 450 W Big Springs Road, Riverside, CA 92507 USA; 2 College of Natural and Agricultural Sciences, University of California Riverside, 900 University Avenue, Riverside, CA 92521 USA; 3 College of Agriculture and Applied Sciences (CAAS), Utah State University (USU), Logan, UT 8432 USA

**Keywords:** Agricultural genetics, Plant genetics

## Abstract

The almond crop has high economic importance on a global scale, but its sensitivity to salinity stress can cause severe yield losses. Salt-tolerant rootstocks are vital for crop economic feasibility under saline conditions. Two commercial rootstocks submitted to salinity, and evaluated through different parameters, had contrasting results with the survival rates of 90.6% for ‘Rootpac 40’ (tolerant) and 38.9% for ‘Nemaguard’ (sensitive) under salinity (Electrical conductivity of water = 3 dS m^−1^). Under salinity, ‘Rootpac 40’ accumulated less Na and Cl and more K in leaves than ‘Nemaguard’. Increased proline accumulation in ‘Nemaguard’ indicated that it was highly stressed by salinity compared to ‘Rootpac 40’. RNA-Seq analysis revealed that a higher degree of differential gene expression was controlled by genotype rather than by treatment. Differentially expressed genes (DEGs) provided insight into the regulation of salinity tolerance in *Prunus*. DEGs associated with stress signaling pathways and transporters may play essential roles in the salinity tolerance of *Prunus*. Some additional vital players involved in salinity stress in *Prunus* include *CBL10*, *AKT1*, *KUP8*, *Prupe.3G053200* (chloride channel), and *Prupe.7G202700* (mechanosensitive ion channel). Genetic components of salinity stress identified in this study may be explored to develop new rootstocks suitable for salinity-affected regions.

## Introduction

After drought, the most severe challenge facing agriculture and crop production is salinization of soil and water resources. Global climate change has led to prolonged and severe droughts in many parts of the world^1^, forcing growers to rely on low-quality water sources and utilize unsustainable irrigation and fertilization practices that increase soil salinization^2^. Saline soil disrupts plant homeostasis in many ways. For example, increases in soil salinity can cause changes in water potential that result in osmotic stress in plants^2^. If prolonged, excess soil salinity leads to ionic stress, macro and micronutrients imbalance, disruption of metabolic processes, and the production of toxic reactive oxidative species (ROS) in salt-stressed plants^3^. These and other salinity effects contribute to decreased productivity^4^. It has been estimated that salinization may impact as much as 50% of arable land by 2050, and worldwide economic losses from salinity stress are estimated to be over tens of billions of U.S. dollars per year^5^. Thus, thorough knowledge of plant salinity tolerance is warranted to maintain crop production that will support both economic growth and food supply.

Numerous studies utilizing model plants have been carried out to uncover molecular mechanisms of plant response to salinity stress. In general, plants activate a signaling cascade in response to salt stress leading to changes in the expressions of genes involved in various biochemical and physiological processes^3,6^. Some specific genes involved in salinity stress have been discovered. For instance, the salt overly sensitive (SOS) gene family members prevent Na^+^ accumulation in plants, leading to increased salt tolerance^7,8^. Other genes of interest include the high-affinity potassium transporter (HKT) family members that aids in preventing root-to-shoot Na^+^ movement, and sodium-hydrogen exchanger (NHX) transporters, found in the vacuoles of plant cells and that aid in maintaining ion homeostasis^9^. Though these findings provide a general foundation for studying plant salinity tolerance, it is noted that plant response to salinity stress, including gene expression, varies considerably among species^10^ and within species^11,12^. Information on these specific cases is scarce for non-model organisms, which is problematic as the effects of salinity on model plants may not entirely reflect the response of other plants^10^.

Almond (*Prunus dulcis*) is an economically important crop in the United States, with over 80% of the world’s almond production coming from California and contributing to over 21.5 billion dollars of California's economy in 2014^13^. Besides their commercial value, almonds are also known for their many nutritional and health benefits^14,15^. However, the almond crop has the highest water footprint among major California crops on a per-unit and aggregate basis^16^. During the past drought in southern California, almond trees had to be eliminated because of insufficient irrigation water. The recurrent drought and excess heat brought by climate change indicate that the use of alternative waters, such as treated municipal wastewater (recycled water), provides a renewable alternative for irrigation with dwindling groundwater. However, recycled waters have a higher salt concentration than freshwater, representing a challenge for their use in glycophytic C3 species sensitive to salinity stress, such as almonds^17^. Root anatomical, cellular, and molecular traits have been investigated in three different almond rootstocks to study mechanisms of salinity tolerance at an early growth stage^18^. It has been shown that increasing electrical conductivity (EC) of experimental irrigation waters led to detrimental effects on the productivity of almonds, including decreased chlorophyll fluorescence, root and shoot growth^19^. Irrigation water with an electrical conductivity (EC_w_) above 1.5 dS m^−1^ affected almond development and growth, and EC_w_ above 4 dS m^−1^ reduced growth by half^20^. The effects of excess salts also depend on the ionic composition of waters, not just the total salinity. When subjected to five different saline water treatments, almond rootstocks grown under sodium/chloride-dominant waters had the highest reduction in survival rate and trunk diameter^21^.

Rootstocks are critical for the success of any almond production system. Rootstocks provide a way to grow different almond cultivars in various soil conditions and mitigate specific stresses^21^. For example, the most widely used rootstock ‘Nemaguard’, a peach-based rootstock, is known for its high yields and resistance to root nematodes^22^. Previously, it was shown that the ‘Nemaguard’ *HKT1* ortholog could restore salinity tolerance in transgenic Arabidopsis *hkt1* mutants^23^. Varying levels of expression of more than 20 salt-stress responsive genes associated with Na^+^ homeostasis (e.g. *SOS1*), K^+^ homeostasis (e.g. *AKT1*), and Cl^−^ homeostasis (e.g. *SLAH1*) have been reported from a large-scale study involving over 14 different rootstocks and their response to salinity stress^21^. Although some individual genes are shown to be critical to salinity stress, the transcriptomic and metabolic effects of salinity stress are poorly understood in *Prunus*. Bioinformatic tools and techniques such as whole-genome sequencing and reference genomes have recently become readily available for the genus *Prunus*^24–26^. RNA-seq approach has been employed to study gene expression in response to drought and salinity in the roots of *Prunus mahaleb* and *Prunus persica*^27^. The dynamics of gene expression in leaves in response to alkaline stress has been reported in *Prunus triloba* Lindl^28^. In addition, water use efficiency-related genes have been identified in the roots of the almond-peach rootstock ‘Garnem’ by employing time-course transcriptome analysis^29^. However, a comparative transcriptomic study of leaves and roots in response to salinity in contrasting *Prunus* rootstocks is lacking.

Previously, we conducted a preliminary screen of several almond rootstocks to evaluate their salinity tolerance and identified ‘Rootpac 40’ as the most salt-tolerant rootstock and ‘Nemaguard’ as the most salt-sensitive (data not shown). The goals of this study were to evaluate the salinity tolerance of two contrasting rootstock cultivars, ‘Rootpac 40’ and ‘Nemaguard’, by examining the effects of salinity on their performance through survival rate and growth; biochemical responses (proline content, antioxidant capacity, total phenolics); physiological responses (chlorophyll content, net photosynthetic rate, and leaf stomatal conductance); mineral element accumulation in leaves; and salinity-induced differential gene expression.

## Results

### Salinity tolerance of ‘Rootpac 40’ and ‘Nemaguard’

**Figure 1:**
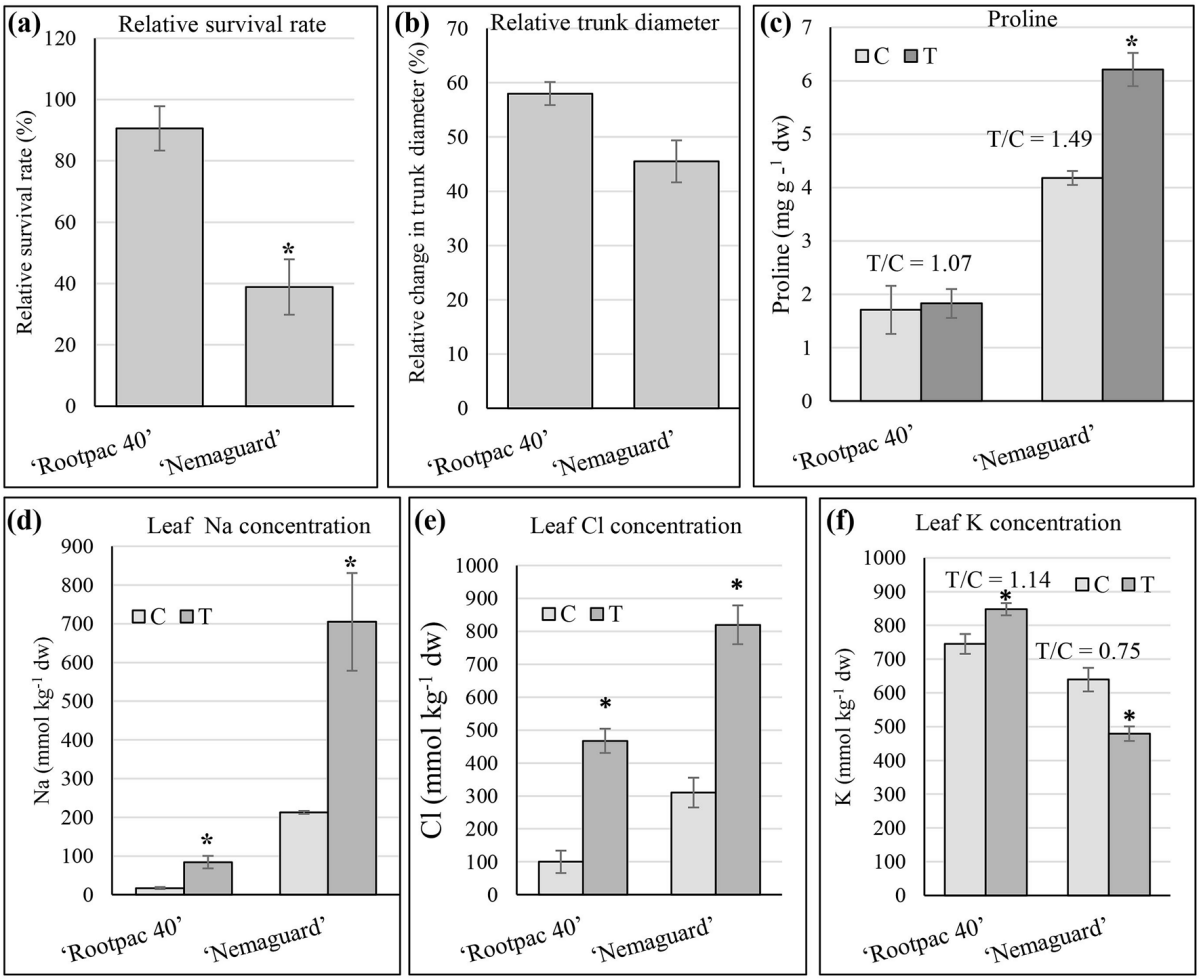
Performances of ‘Rootpac 40’ and ‘Nemaguard’ rootstocks under control and saline environments. (**a**) Relative survival rates (%) of ‘Rootpac 40’ and ‘Nemaguard’ rootstocks in response to the saline treatment compared to control. (**b**) Relative change in trunk diameters. (**c**) Leaf proline concentrations. (**d**) Leaf Na concentrations. (**e**) Leaf Cl concentrations. (**f**) Leaf K concentrations. Error bars represent standard errors of three biological replicates. An asterisk (*) indicates a significant difference (t-test *p* ≤ 0.05). C indicates control, and T indicates saline treatment.

The survival rate analysis revealed a higher survival rate for ‘Rootpac 40’ (90.6%) compared to ‘Nemaguard’ (38.9%) (Fig. 1a). The relative percent change of ‘Rootpac 40’ trunk diameter (58%) was slightly greater (*p*-value = 0.06) than that of ‘Nemaguard’ (45.5%) (Fig. 1b).

### Effect of salinity on biochemical responses

Proline concentration, antioxidant capacity (oxygen radical absorbance capacity, ORAC), and total phenolics in leaves of ‘Rootpac 40’ and ‘Nemaguard’ were evaluated under control and saline treatments (Fig. 1c and Supplemental Fig. S1). Our data indicated a comparable proline accumulation under both control and salinity treatments for ‘Rootpac 40’, while ‘Nemaguard’ had not only a higher leaf proline concentration, but also a significantly higher increase in proline concentration than ‘Rootpac 40’ in response to salinity (Fig. 1c). There were no significant changes in antioxidant capacity in either ‘Nemaguard’ or ‘Rootpac 40’ in response to salinity treatment (Supplementary Fig. S1a). Similarly, neither ‘Nemaguard’ nor ‘Rootpac 40’, showed significant differences for total phenolics in response to salinity treatment (Supplementary Fig. S1b).

### Effect of salinity on gas exchange parameters

To study how salt stress affects gas exchange parameters in ‘Rootpac 40’ and ‘Nemaguard’ rootstocks, we evaluated chlorophyll content by Soil–Plant Analysis Development (*SPAD*) analysis, net photosynthetic rate (*Pn*), and leaf stomatal conductance (*gs*). Although *SPAD* readings resulted in no significant effect of salinity on chlorophyll content on either rootstock, the average SPAD value was slightly smaller in ‘Nemaguard’ than in ‘Rootpac 40’ in both control and salt-treated rootstocks (Supplementary Fig. S2a). The photosynthetic efficiency (*Pn*) value analysis of ‘Nemaguard’ and ‘Rootpac 40’ indicated that salinity significantly inhibited photosynthesis in leaves of both rootstocks (Supplementary Fig. S2b). The stomatal conductance (*gs*) data revealed that salinity reduced stomatal conductance in both rootstocks, although not significantly for ‘Nemaguard’, which had a high variation in *gs* values compared to ‘Rootpac 40’ (Supplementary Fig. S2c). However, it should be noted that, under salinity, ‘Rootpac 40’ and ‘Nemaguard’ had similar stomatal conductance values (Supplementary Fig. S2c).

### Leaf mineral element accumulation in ‘Rootpac 40’ and ‘Nemaguard’ in response to salinity

To study ion accumulation characteristics of ‘Rootpac 40’ and ‘Nemaguard’ in response to salinity stress, mineral element analysis was performed on leaf samples for Na, Cl, and K (Fig. 1d–f) and for Ca, Mg, P, S, B, Cu, Fe, Mn, Mo, and Zn (Supplementary Fig. S3). Both rootstocks showed a higher accumulation of Na compared to their corresponding control in response to salinity stress. However, in response to salinity, ‘Nemaguard’ leaves accumulated over eight times the concentration of Na than that found in ‘Rootpac 40’ leaves (Fig. 1d). Similar to Na accumulation, both genotypes, ‘Rootpac 40’ and ‘Nemaguard’, showed significantly higher accumulations of leaf Cl under salinity than under control. The average accumulation of Cl in ‘Nemaguard’ leaves was 1.75-fold higher than that of ‘Rootpac 40’ (Fig. 1e). It is worth noting that under the control condition, ‘Rootpac 40’ showed 12-fold less Na accumulation and three-fold less Cl accumulation than ‘Nemaguard’. Additionally, a significant increase in K accumulation was observed in ‘Rootpac 40’ in response to salinity, showing a treatment/control (T/C) K accumulation ratio of 1.14. However, a significant decrease in K accumulation in response to salt treatment was observed in ‘Nemaguard’ leaves with a T/C ratio of 0.75 (Fig. 1f). There was no significant accumulation of leaf Ca, Mg, S, B, Cu, Fe, Mn, Mo, or Zn between ‘Nemaguard’ and ‘Rootpac 40’ in response to salinity (Supplementary Fig. S3). Although significant, the decrease in leaf P of ‘Rootpac 40’ was very small. ‘Nemaguard’ maintained a similar leaf P accumulation under control and salinity conditions (Supplementary Fig. S3c).

### Transcript sequencing and gene expression

Leaf and root transcriptomes of salt-tolerant ‘Rootpac 40’ and salt-sensitive ‘Nemaguard’ rootstocks were studied under control and salinity. We named our experimental samples as CNL for Control ‘Nemaguard’ Leaf, TNL for Treatment ‘Nemaguard’ Leaf, CNR for Control ‘Nemaguard’ Root, TNR for Treatment ‘Nemaguard’ Root, CRL for Control ‘Rootpac 40’ Leaf, TRL for Treatment ‘Rootpac 40’ Leaf, CRR for Control ‘Rootpac 40’ Root, and TRR for Treatment ‘Rootpac 40’ Root (Supplementary Table S1). We observed a total of 1,586,189,238 raw reads for 24 libraries with an average of 66,091,218 raw reads/library (Supplementary Table S2). After removing adapter sequences, low-quality reads, and ambiguous nucleotides, we obtained 1,551,884,576 clean reads, consisting of 233 gigabases (Gb) with an average of 9.7 Gb per library (Supplementary Table S2).

**Figure 2:**
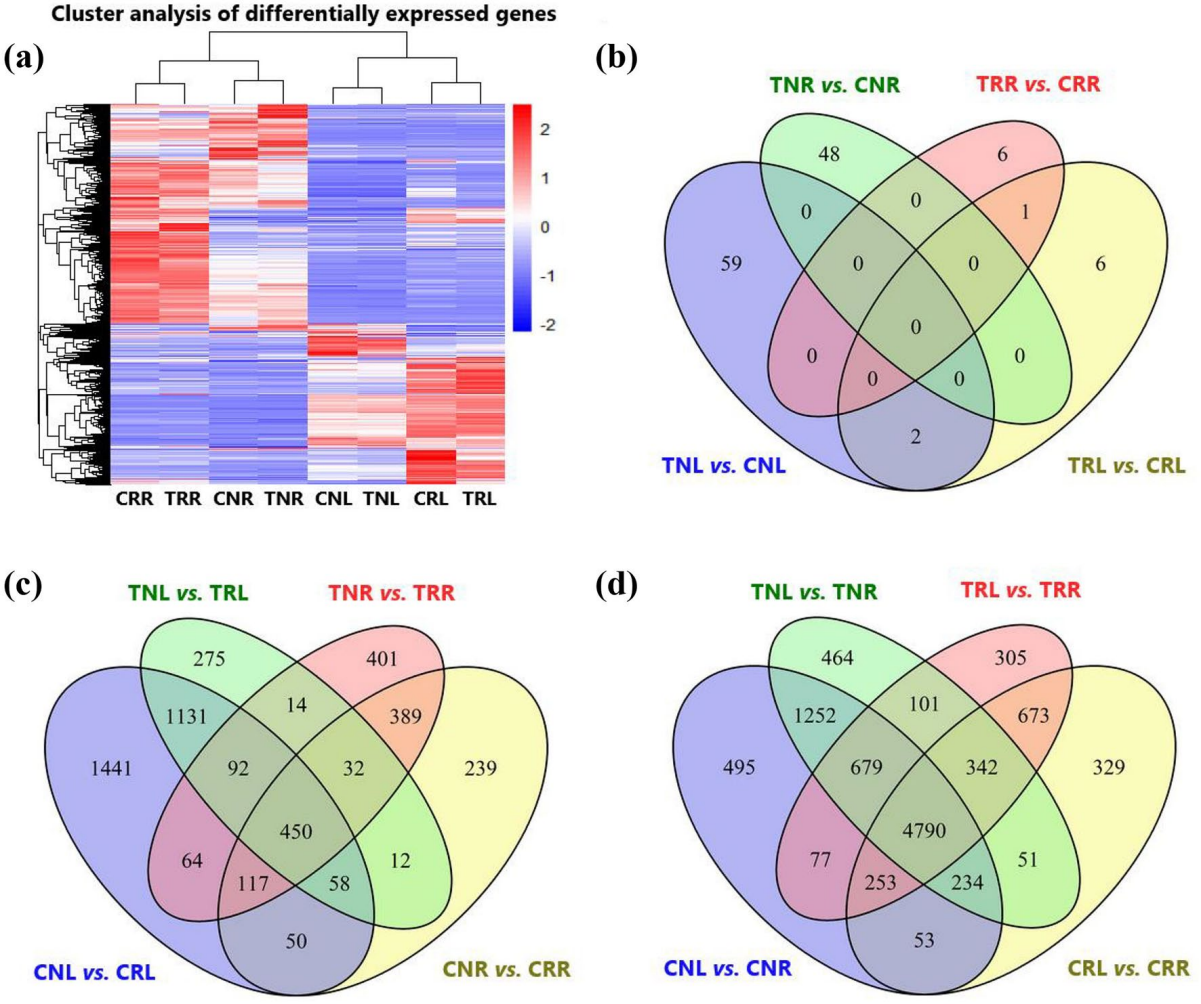
Heat map-based clustering and Venn diagram analysis of differentially expressed genes (DEGs). (**a**) Heatmap and hierarchical clustering showing the status of gene expression and clustering of DEGs (those which were differentially expressed at least in one comparison) across all eight indicated samples in a specific column. (**b**) Venn diagram shows the number of DEGs across four salt treatment versus control comparisons. (**c**) Venn diagram shows the number of DEGs across four salt-sensitive (‘Nemaguard’) versus four salt-tolerant (‘Rootpac 40’) comparisons. (**d**) Venn diagram shows the number of DEGs across four leaf versus root comparisons as indicated from left to right. Compared samples are shown from left to right in (**b**)–(**d**). CNL, Control ‘Nemaguard’ Leaf; TNL, Treatment ‘Nemaguard’ Leaf; CNR, Control ‘Nemaguard’ Root; TNR, Treatment ‘Nemaguard’ Root; CRL, Control ‘Rootpac 40’ Leaf; TRL, Treatment Rootpac Leaf; CRR: control ‘Rootpac 40’ root; TRR, treatment ‘Rootpac 40’ Root.

To analyze differential gene expression, RNA-Seq reads from each sample were aligned to the annotated *Prunus persica* genome, which produced an average mapping of 92.27% for individual samples (Supplementary Table S2). The genome sequencing of *P. persica* predicted 27,852 protein-coding genes^26^. Our analyses identified 14,985 DEGs in at least one of the comparisons: Treatment versus control, ‘Nemaguard’ versus ‘Rootpac 40’, or Leaf versus Root (Fig. 2 and Table 1). Gene expression-based cluster analysis identified two main groups based on tissue types, one for root tissues and the other for leaf tissues (Fig. 2a). Genes from CRR and TRR formed one subgroup, and genes from CNR and TNR formed the other subgroup within the root and similar observations were made in the leaf groups (Fig. 2a).

**Table 1:** Differentially Expressed Genes (DEGs) identified in different comparisons.

Comparison	Groups	DEGs	Upregulated	Downregulated
Salt versus control	TNL versus CNL	61	4	57
	TNR versus CNR	48	30	18
	TRL versus CRL	9	1	8
	TRR versus CRR	7	1	6
‘Nemaguard’ versus ‘Rootpac 40’	CNL versus CRL	3403	1338	2065
	CNR versus CRR	1347	542	805
	TNL versus TRL	2064	799	1265
	TNR versus TRR	1559	706	853
Leaf versus root	CNL versus CNR	7853	3456	4377
	CRL versus CRR	6725	3211	3514
	TNL versus TNR	7913	3231	4682
	TRL versus TRR	7220	3267	3953

CNL, control ‘Nemaguard’ leaf; TNL, treatment ‘Nemaguard’ leaf; CNR, control ‘Nemaguard’ root, TNR, treatment ‘Nemaguard’ root; CRL, control ‘Rootpac 40’ leaf, TRL, treatment ‘Rootpac 40’ leaf; CRR, control ‘Rootpac 40’ root; TRR, treatment ‘Rootpac 40’ root.

Gene expression analyses were performed to identify DEGs in three different comparisons (treatment versus control; salt-sensitive rootstock versus salt-tolerant rootstock; and leaf versus root) (Fig. 2, Table 1 and Supplementary Table S3). For the treatment versus control comparisons, 122 DEGs were identified, including 61 for TNL versus CNL (4 upregulated and 57 downregulated), 48 for TNR versus CNR (30 upregulated and 18 downregulated), 9 for TRL versus CRL (1 upregulated and 8 downregulated), and 7 for TRR versus CRR (1 upregulated and 6 downregulated) (Fig. 2b, Table 1 and Supplementary Table S3). For the comparisons between salt-sensitive (‘Nemaguard’) versus salt-tolerant (‘Rootpac 40’) rootstocks, 4765 DEGs were identified, including 3403 for CNL versus CRL (1338 upregulated and 2065 downregulated), 1347 for CNR versus CRR (542 upregulated and 805 downregulated), 2064 for TNL versus TRL (799 upregulated and 1265 downregulated), and 1559 for TNR versus TRR (706 upregulated and 853 downregulated) (Fig. 2c, Table 1 and Supplementary Table S3). For the comparisons between leaf versus root, 10,098 DEGs were identified, including 7833 in CNL versus CNR (3456 upregulated and 4377 downregulated), 6725 in CRL versus CRR (3211 upregulated and 3514 downregulated), 7913 in TNL versus TNR (3231 upregulated and 4682 downregulated) and 7220 in TRL versus TRR (3267 upregulated and 3953 downregulated) (Fig. 2d, Table 1 and Supplementary Table S3). 

### Verification of DEGs using qRT-PCR

**Figure 3:**
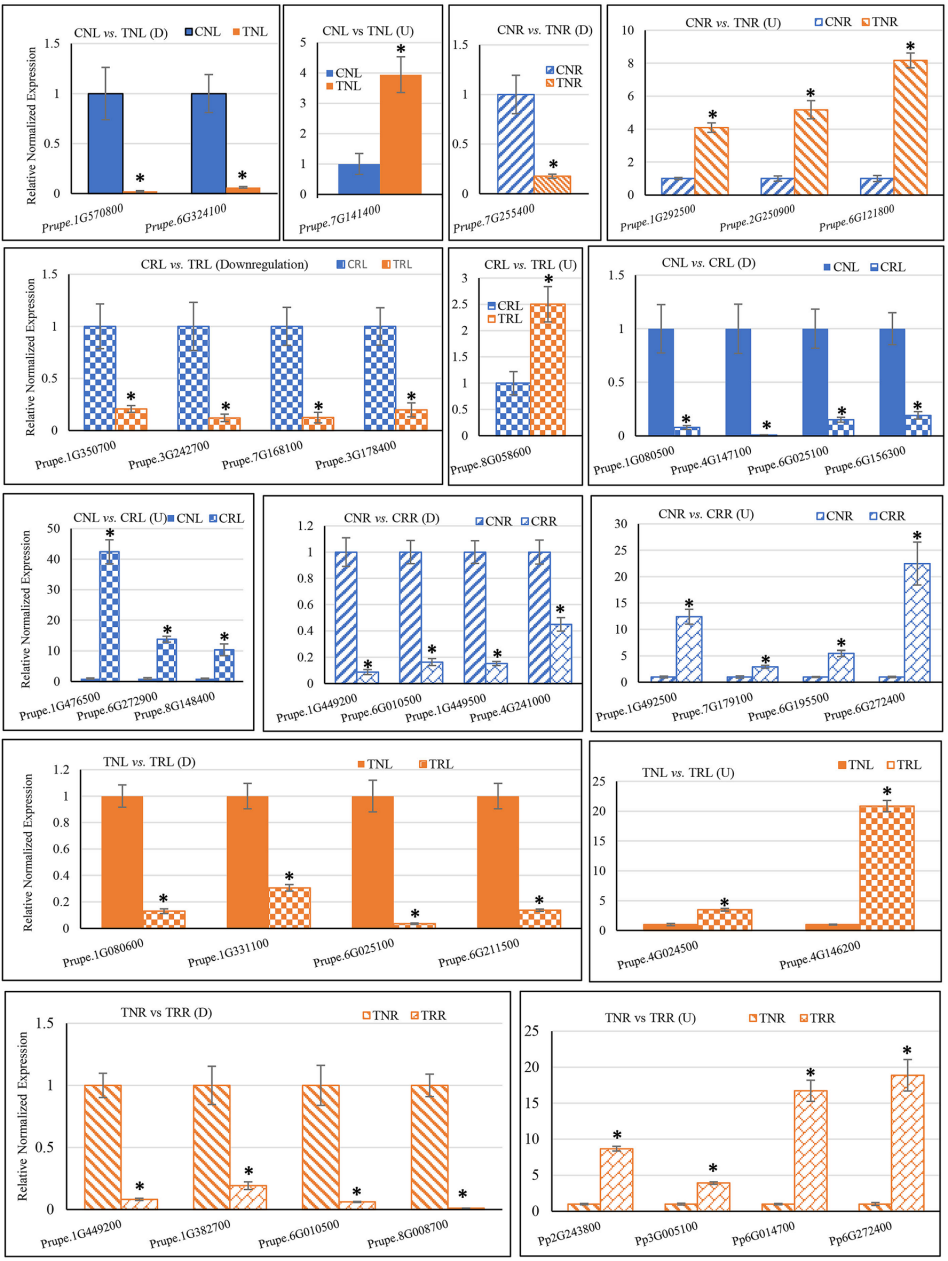
qRT-PCR validation of gene expression observed in RNA-Seq data. The *Y*-axis indicates relative normalized expression, and the *X*-axis indicates gene IDs. Samples used for the gene expression comparisons are shown on top of the graph. Graphs for downregulated (D) gene(s) and upregulated (U) genes are shown separately. The blue color indicates control (blue solid, CNL; blue diagonal stripes, CNR; blue large checkerboard, CRL; blue diagonal bricks, CRR), and the orange color indicates treatment (orange solid, TNL; orange diagonal stripes, TNR; orange large checkerboard TRL; and orange diagonal bricks, TRR). An asterisk (*) indicates a significant difference (t-test *p* ≤ 0.05). CNL, Control ‘Nemaguard’ Leaf; TNL, Treatment ‘Nemaguard’ Leaf; CNR, Control ‘Nemaguard’ Root; TNR, Treatment ‘Nemaguard’ Root; CRL, Control ‘Rootpac 40’ Leaf; TRL, Treatment Rootpac Leaf; CRR: control ‘Rootpac 40’ root; TRR, treatment ‘Rootpac 40’ Root.

To validate RNA-Seq data, we randomly selected a total of 41 DEGs (18 upregulated and 23 downregulated genes) from different comparison groups to perform qRT-PCR (Supplementary Tables S4 & S5). Among the 41 DEGs, 33 genes were evaluated for single comparisons, and four were evaluated for two different comparisons. Relative normalized expressions data were compared for three genes for CNL versus TNL; four genes for CNR versus TNR; five genes for CRL versus TRL; seven genes for CNL versus CRL; eight genes for CNR versus CRR; six genes for TNL versus TRL; and eight genes for TNR versus TRR (Fig. 3). For most genes, qRT-PCR assay results showed a general trend of expression profiles observed in the RNA-Seq experiment, confirming the validity of the RNA-Seq results (Fig. 3 & Supplementary Fig. S4). For example, *Prupe.8G148400,* which encodes a serine carboxypeptidase showed 8.9- and 10.3-fold upregulation in CRL compared to CNL, in RNA-Seq and qRT-PCR, respectively. Still, a few genes did not exhibit a similar fold change in qRT-PCR compared to RNA-Seq results. For example, *Prupe.1G476500,* which encodes a BURP protein, showed 95.3- and 42.3-fold upregulation in CNL compared to CRL, in RNA-seq and qRT-PCR, respectively. Although expression levels differed between RNA-Seq and qRT-PCR results for a few genes, overall trends (downregulation or upregulation) were the same.

### Gene ontology (GO) enrichment analysis of DEGs

To study functional enrichment analysis of DEGs in treatment versus control and ‘Nemaguard’ versus ‘Rootpac 40’, we performed GO enrichment analysis primarily for three major categories: molecular function, MF; cellular component, CC; and biological processes, BP (Supplementary Table S6). In treatment versus control comparisons, 111, 86, 20, and 31 GO terms were enriched in TNL versus CNL, TNR versus CNR, TRL versus CRL, and TRR versus CRR, respectively (Supplementary Table S6). In ‘Nemaguard’ versus ‘Rootpac 40’ comparison, 1552, 901, 1145, and 931 GO terms were enriched in CNL versus CRL, CNR versus CRR, TNL versus TRL, and TNR versus TRR, respectively (Supplementary Table S6).

### KEGG enrichment analysis of DEGs

To find out which biological pathways were enriched in treatment versus control and ‘Nemaguard’ (salt-sensitive) versus ‘Rootpac 40’ (salt-tolerant), KEGG enrichment analysis of DEGs was performed for each pairwise comparison. In salt treatment versus control comparisons, 1, 4, 0, and 1 pathway(s) were enriched in TNL versus CNL, TNR versus CNR, TRL versus CRL, and TRR versus CRR, respectively (Supplementary Table S7). In salt-sensitive (‘Nemaguard’) versus salt-tolerant (‘Rootpac 40’) comparisons, 7, 5, 3, and 4 pathways were enriched in CNL versus CRL, CNR versus CRR, TNL versus TRL, and TNR versus TRR, respectively (Supplementary Table S7).

### DEGs associated with stress pathways

**Figure 4:**
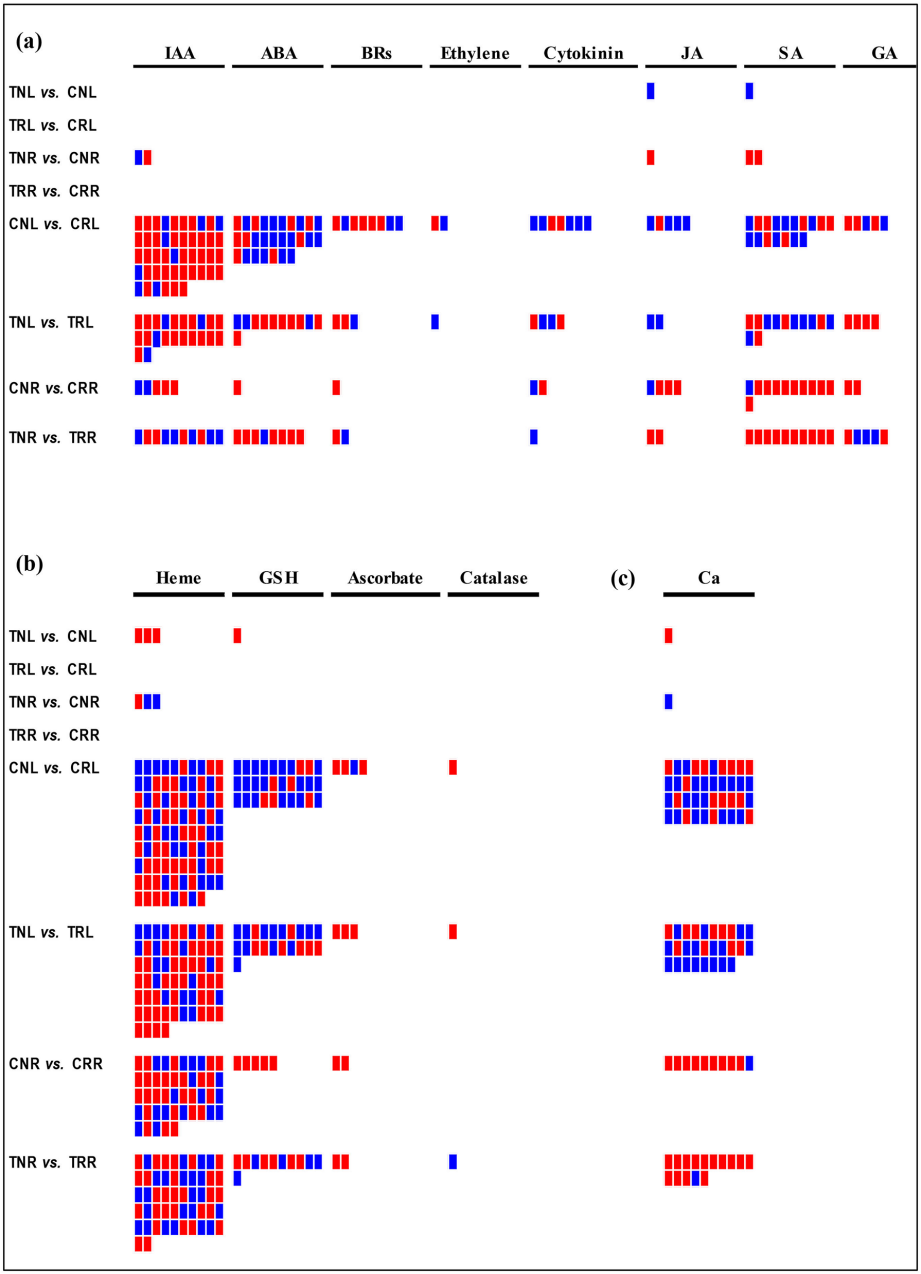
DEGs associated with phytohormone, redox, and calcium signaling pathways. (**a**) DEGs associated with phytohormone pathways (are indicated at the top). (**b**) DEGs associated with redox signaling pathways (are indicated at the top). (**c**) DEGs associated with calcium signaling (Ca). The red color indicates downregulated DEGs, and the blue color indicates upregulated DEGs. Left-side texts show pairwise comparisons. The top four compared samples indicate treatment versus control, and the bottom for compared samples indicate salt-sensitive versus salt-tolerant comparisons. *IAA*, indole acetic acid (auxin); *ABA*, abscisic acid; *BRs*, brassinosteroids; *JA*, jasmonic acid; *SA*, salicylic acid; *GA,* gibberellins; *GSH*, glutathione; *Ca*, calcium.

Multiple pathways regulate salt stress signaling in plants. Therefore, all identified DEGs were analyzed to determine their association with primary stress pathways such as phytohormone signaling, redox signaling, and calcium signaling (Fig. 4 and Supplementary Tables S8, S9, and S10).

#### Hormonal signaling

In treatment versus control comparisons, two DEGs were upregulated, 1 for jasmonic acid (JA) and 1 for salicylic acid (SA) in TNL versus CNL (Fig. 4 and Supplementary Table S8). In TNR versus CNR, 5 DEGs were associated with hormonal signaling, including 2 DEGs (one upregulated and one downregulated) for indole acetic acid (IAA), 1 downregulated DEG for JA, and 2 downregulated DEGs for SA. No DEGs were identified for hormonal signaling in the two comparisons TRL versus CRL and TRR versus CRR (Fig. 4 and Supplementary Table S8). In salt-sensitive versus salt-tolerant comparisons, we identified 117, 26, 59, and 37 DEGs associated with hormone signaling for the comparisons CNL versus CRL, CNR versus CRR, TNL versus TRL, and TNR versus TRR, respectively (Fig. 4a and Supplementary Table S8). The highest number of DEGs involved in hormonal signaling were identified for IAA, followed by SA and ABA.

#### Redox signaling

In treatment versus control comparisons, 4 DEGs associated with redox signaling were downregulated in TNL versus CNL; of these, 3 DEGs were for heme, and 1 DEG was for glutathione (GSH) (Fig. 4b and Supplementary Table S9). Three DEGs (1 upregulated and two downregulated) were involved in redox signaling, specifically associated with heme in TNR versus CNR. No DEGs associated with redox signaling were identified for TRL versus CRL and TRR versus CRR comparisons (Fig. 4b and Supplementary Table S9). In salt-sensitive versus salt-tolerant comparisons, 123, 64, 52, and 76 DEGs regulating redox pathways were identified for the CNL versus CRL, TNL versus TRL, CNR versus CRR, TNR versus TRR comparisons, respectively (Fig. 4b and Supplementary Table S9). The highest number of DEGs associated with redox signaling were identified for heme, followed by GSH.

#### Calcium signaling

In the salt treatment versus control comparisons, one DEG was downregulated in TNL versus CNL, and one DEG was upregulated in TNR versus CNR (Fig. 4c and Supplementary Table S10). No DEGs were involved in calcium signaling in the TRL versus CRL and TRR versus CRR comparisons (Fig. 4c and Supplementary Table S10). In salt-sensitive versus salt-tolerant comparisons, 40, 27, 10, and 14 DEGs involved in calcium signaling were identified for the CNL versus CRL, TNL versus TRL, CNR versus CRR, TNR versus TRR comparisons, respectively (Fig. 4c and Supplementary Table S10).

### DEGs associated with transporters

**Figure 5:**
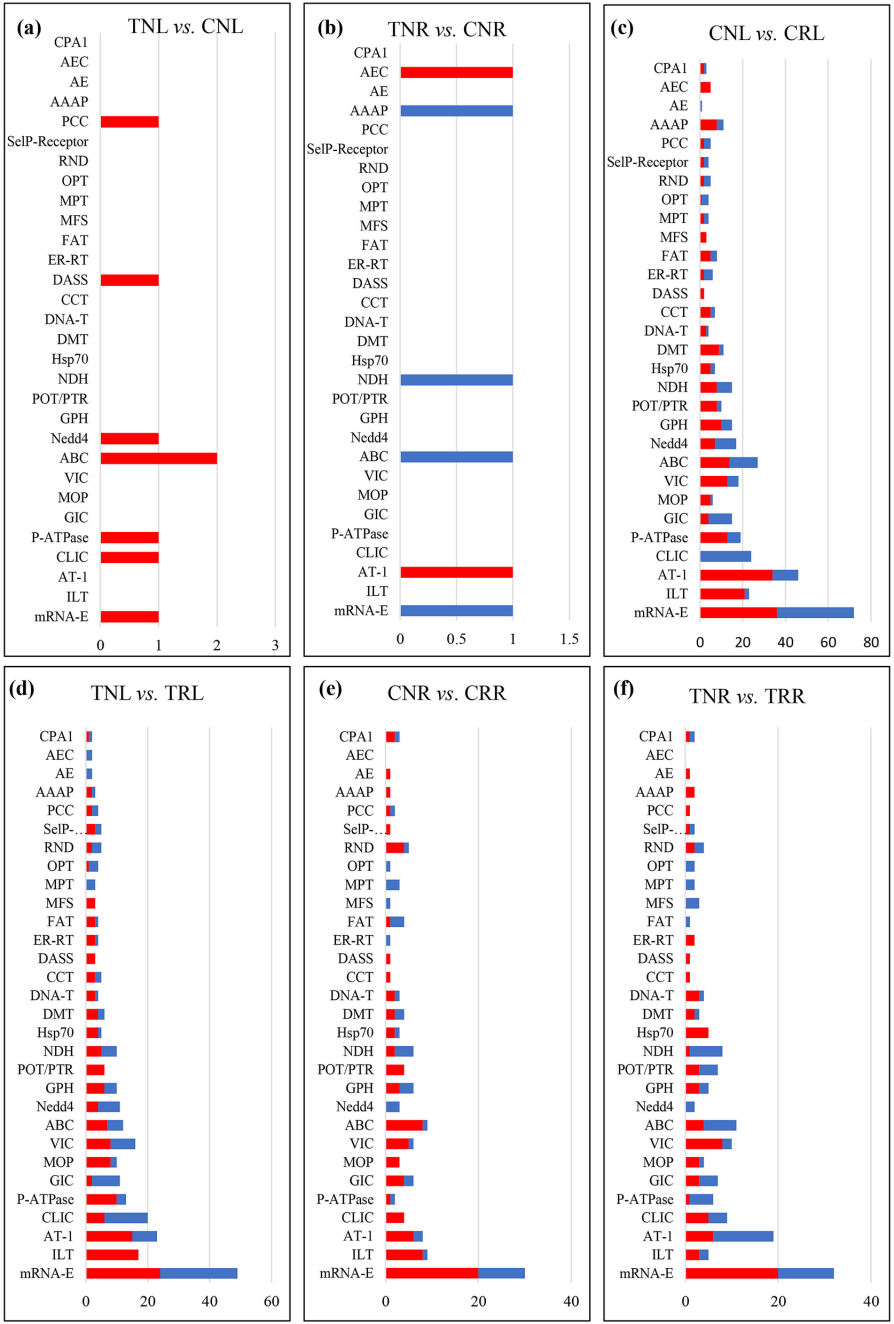
Transporter analysis of DEGs. The *Y*-axis indicates transporter superfamilies. The *X*-axis shows gene counts. Salt treatment versus control comparisons are shown in (**a**) and (**b**). Salt-sensitive versus salt-tolerant comparisons are shown in (**c**)–(**f**). The red color indicates downregulated DEGs, and the blue color indicates upregulated DEGs. Compared samples are indicated at the top of each panel.

Transporters play critical roles in ion distribution and homeostasis in plants. Our observations indicated that the salinity tolerance differences between ‘Rootpac 40’ and ‘Nemaguard’ were primarily due to contrasting accumulation of Na, Cl, and K. This prompted us to analyze DEGs that encode transporters. Our analysis identified a total of 1194 transporter DEGs in treatment versus control comparisons and salt-sensitive versus salt-tolerant comparisons (Fig. 5 and Supplementary Table S11).

In TNL versus CNL comparison, 8 DEGs were found encoding 7 families of transporters, including the ATP-binding cassette (ABC) superfamily, the divalent anion Na^+^ symporter (DASS) family, and the intracellular chloride channel (CLIC) family. In TNR versus CNR, 6 DEGs were identified, encoding 6 families of transporters, including the ATP-binding cassette (ABC) superfamily and the autotransporter-1 (AT-1) family (Fig. 5 and Supplementary Table S11). No DEGs were identified encoding transporter families in TRL versus CRL and TRR versus CRR comparisons. In salt-sensitive versus salt-tolerant comparisons, 488 DEGs were found encoding 76 transporter families in CNL versus CRL, including the intracellular chloride channel (CLIC) family, the voltage-gated ion channel (VIC) superfamily, and the monovalent cation:proton antiporter-1 (CPA1) family. For TNL versus TRL, 319 DEGs were found encoding 63 families of transporters, including the polycystin cation channel (PCC) family, the cation channel-forming heat shock protein-70 (Hsp70) family, the Glycoside-Pentoside-Hexuronide (GPH): cation symporter family, the calcium-dependent chloride channel (Ca-ClC) family and the K^+^ uptake permease (KUP) family. In CNR versus CRR, 170 DEGs encoded 54 transporter families, including the voltage-gated K^+^ channel ÃŽÂ^2^-subunit (KvÃŽÂ^2^) family, the anion exchanger (AE) family, and the proton-dependent oligopeptide transporter (POT/PTR) family. In TNR versus TRR, 203 DEGs encoded 58 transporter families, including the DASS family and the autotransporter-1 (AT-1) family (Fig. 5 and Supplementary Table S11). Our analyses also found DEGs that encode for similar transporters across comparisons (Fig. 5 and Supplementary Table S11).

## Discussion

We compared the rootstocks ‘Rootpac 40’ with ‘Nemaguard’ under control and salinity conditions for relative survival rate, relative growth rate (specifically trunk diameter), leaf mineral element concentration, accumulation of proline, antioxidant capacity, total phenolics accumulation, chlorophyll content, net photosynthesis, stomatal conductance, and global gene expression by performing RNA-Seq analysis.

The comparison of ‘Rootpac 40’ and ‘Nemaguard’ for various parameters established a relatively higher salinity tolerance of ‘Rootpac 40’ compared to ‘Nemaguard’. First, in response to salinity treatment, ‘Rootpac 40’ showed higher relative survival rate and better growth than ‘Nemaguard’ (Fig. 1a and b). Second, ‘Rootpac 40’ rootstock accumulated less Na compared to ‘Nemaguard’ in leaf tissue (Fig. 1d), indicating that ‘Rootpac 40’ has the better ability to control Na accumulation in leaf during salinity stress. Third, ‘Rootpac 40’ showed less Cl accumulation than ‘Nemaguard’ in response to salinity treatment indicating that ‘Rootpac 40’ may take less Cl from soil and/or possess better Cl^−^ exclusion mechanism (Fig. 1e). Fourth, ‘Rootpac 40’ showed a higher accumulation of K in response to salinity treatment than the control, whereas ‘Nemaguard’ showed a reduced accumulation of K in response to salinity treatment than control (Fig. 1f). These observations are in line with a previous study, where screening of 14 almond rootstocks with different genetic backgrounds revealed that peach-almond hybrids (such as ‘Rootpac 40’) transport less Na, less Cl, and more K to leaves than peach-based rootstocks (such as ‘Nemaguard’) and have better salinity tolerance^21^.

To find out differences in gene expression in response to salinity stress at the transcriptome level, an RNA-Seq experiment was performed on root and leaf tissues of ‘Nemaguard’ (salt-sensitive) and ‘Rootpac 40’ (salt-tolerant), under the control and salinity treatments (Supplementary Table S1). A higher number of DEGs were detected in salt-sensitive versus salt-tolerant comparisons than treatment versus control comparisons, suggesting that genotype-specific differences are more critical in *Prunus* than treatment-specific differences.

Phytohormones are important signaling components and play critical roles in response to various stresses, including salinity stress^30,31^. Expectedly, a higher number of genes associated with phytohormone signaling were differentially expressed in salt-sensitive versus salt-tolerant genotype comparisons than in treatment versus control comparisons (Fig. 4a & Supplementary Table S8). Our analysis revealed the highest number of DEGs associated with auxin (IAA), followed by SA, and ABA; only a few DEGs were associated with GA, JA, BRs, cytokinin, or ethylene (Fig. 4a & Supplementary Table S8). These observations indicate that auxin, SA, and ABA may play critical signaling roles in response to salinity stress in *Prunus*. Additionally, in salt-sensitive versus salt-tolerant comparisons, a significantly higher number of DEGs were observed in leaves than roots, suggesting a more critical role of hormonal signaling in leaf during salinity stress^32^.

Reactive oxygen species (ROS) function as essential secondary messengers in cellular signaling in responses to various stresses in plants. Over accumulation of ROS (hydrogen peroxide, hydroxyl radicals, and superoxide anions) negatively impacts the function of molecular machinery and cellular structures, and uncontrolled over-accumulation of ROS could lead to cell death^33^. To maintain a non-toxic level of ROS, plants have both non-enzymatic and enzymatic antioxidative systems, which also play important roles in abiotic stress tolerance. Plants have many ROS scavenging enzymes, including glutathione S-transferase, superoxide dismutase, ascorbate peroxidase (APX), monodehydroascorbate reductase, dehydroascorbate reductase, glutathione reductase, peroxiredoxin, and catalase^34^. Plants also produce non-enzymatic antioxidants, including glutathione (GSH), phenolic compounds, tocopherols, and ascorbic acid. In plants, heme-containing proteins like catalase and APX play critical roles in ROS homeostasis^35^. For redox signaling, we identified the highest number of DEGs encoding heme-containing proteins, suggesting critical roles of these proteins in salinity tolerance (Fig. 4b and Supplementary Table S9). Additionally, genes associated with glutathione (GSH), ascorbate, and catalase were also differentially regulated, indicating their roles in ROS homeostasis in response to salinity stress in *Prunus* (Fig. 4b and Supplementary Table S9).

Calcium serves as an important second messenger in cellular signaling, including salinity stress-induced response^36^. In treatment versus control comparisons, only 2 DEGs associated with calcium signaling were identified in ‘Nemaguard’ (Fig. 4c). Nevertheless, in ‘Rootpac 40’ no DEGs were identified associated with calcium signaling. These findings suggest that the expressions of genes associated with calcium signaling are stable under the control and the salinity treatments for both rootstocks. In contrast, more than 90 DEGs were identified in salt-sensitive versus salt-tolerant comparisons, indicating that the expression differences in Ca^2+^ signaling genes between two genotypes may explain differences in their salinity tolerance (Fig. 4c).

Calcium signaling plays a key role in salinity tolerance in plants by activating the SOS signaling pathway, which is critical for Na homeostasis^37,38^. In addition to SOS3 (aka CBL4), CBL10 serves as a calcium sensor during salinity stress^39^. Salinity stress induces intracellular Ca^2+^ concentration; upon sensing Ca^2+^, CBL10 interacts and activates SOS2 (aka CIPK24). Subsequently, SOS2 phosphorylates SOS1 that in turn activates SOS1. Active SOS1 extrudes Na^+^ from the inside of the cell to the outside. *Prupe.1G412900* encodes CBL10*,* which showed much higher expression in leaf and root tissues of ‘Rootpac 40’ than ‘Nemaguard’ irrespective of salinity treatment status (both in control and saline treatment conditions) (Supplementary Table S3). The higher expression of CBL10 in ‘Rootpac 40’ may be critical in maintaining Na^+^ homeostasis, providing higher salinity tolerance compared to ‘Nemaguard’.

In addition to plant growth and development, potassium homeostasis is vital for salinity tolerance. In response to salinity, we observed an increased leaf K accumulation in ‘Rootpac 40’ but decreased in ‘Nemaguard’ (Fig. 2). Potassium channels and transporters play essential roles in K^+^ absorption from the soil. AKT1 (Arabidopsis K^+^ transporter 1) plays an essential role in K^+^ uptake from soil^40^. Our analyses identified a low expression of *AKT1* (*Prupe.1G472600*) in control ‘Nemaguard’ leaf (CNL) compared to the control ‘Rootpac 40’ leaf (CRL) (Supplementary Table S3). Similarly, we also observed a higher expression of *AKT1* in TRL compared to TNL. Additionally, higher expression of *AKT1* was observed in TRR compared to TNR. These facts suggest that higher expression of *AKT1* may have contributed to a higher concentration of K during control and salinity treatment condition in ‘Rootpac 40’. Hence, *AKT1* may be a determinant for salinity tolerance in *Prunus*. Similarly, both under control and treatment conditions (CNL versus CRL and TNL versus TRL), lower expression of *Prupe.5G236500* was observed, which encodes a potassium transporter. Arabidopsis *KUP8* (potassium uptake permease 8) is an ortholog *Prupe.5G236500*. In Arabidopsis, 13 genes, including *KUP8,* belong to the K^+^ uptake family (KUP/HAK/KT), are implicated in potassium transport and translocation^41^. Therefore, higher expression of *Prupe.5G236500* in ‘Rootpac 40’ leaf compared to ‘Nemaguard’ in control and salinity treatment conditions may have contributed to higher accumulation of K^+^ in ‘Rootpac 40’.

We observed a lower accumulation of Cl in ‘Rootpac 40’ compared to ‘Nemaguard’ in response to salinity stress. Our differential gene expression analysis identified a few promising candidate genes. For example, *Prupe.3G053200* encodes anoctamin-10 (ANO10)/ chloride channel. Lower expression of this gene was observed in CNL compared to CRL (Supplementary Table S3). Similarly, lower expression of this gene was observed in TNL compared to TRL. Arabidopsis ortholog of this gene, *AtTMEM16*, is an anion/H^+^ symporter that prefers Cl^−^ over NO_3_^−^, suggesting that *Prupe.3G053200* may have contributed to differential accumulation of Cl in both rootstocks in response to salinity^42^. Functional characterization of *Prupe.3G053200* should reveal its biological role in Cl^−^ homeostasis in response to salinity. Additionally, *Prupe.7G202700*, which encodes a mechanosensitive ion channel, was found to have lower expression in TNL compared to TRL (Supplementary Table S3), which might have contributed to lower accumulation of Cl in ‘Rootpac 40’ compared to ‘Nemaguard’ as it can extrude Cl^−^ from inside the cell to outside^43^.

A plant's ability to tolerate salt has been linked with total phenolics and antioxidant capacity^12,44^. In the present study, neither ‘Rootpac 40’ nor ‘Nemaguard’ showed significant differences between control and salinity for total phenolics or antioxidant capacity resulting from the ORAC test, suggesting that the salinity tolerance of these rootstocks was not directly dependent on total phenolics or antioxidant capacity measured in terms of relative survival rate or leaf mineral element accumulation precisely of Na and Cl (Fig. 1).

Our findings revealed that ‘Nemaguard’ (salt-sensitive) showed significant induction of proline accumulation in response to salinity, whereas ‘Rootpac 40’ (salt-tolerant) did not (Fig. 1C). Higher proline accumulation in the salt-sensitive rootstock may indicate that ‘Nemaguard’, but not ‘Rootpac 40’, was under an increased stress level resulting from the applied salinity of EC_w_ = 3.0 dS m^−1^. Accordingly, a previous comparative analysis between 14 almond rootstocks revealed that in response to 10-month salinity treatment, rootstocks that accumulated less proline were more salt-tolerant than rootstocks that accumulated higher proline^21^. Also, the same report described that the treatment/control (T/C) ratio of proline accumulation had an inverse correlation with survival rate and positive correlation for Na or Cl accumulation in leaf and higher salinity tolerance ability^21^, which agrees with the proline salt-response values observed in the present study (Fig. 1). These facts suggest that the amount of proline accumulation in *Prunus* genotypes depends on the amount of stress they sense. Under saline conditions, salt-sensitive genotypes are under higher stress than salt-tolerant genotypes; hence they accumulate more proline. It has also been suggested that proline accumulation is an indicator of salinity stress in real agricultural settings^21^.

In addition to the different mechanisms discussed above, our analysis identified several candidate genes that also showed extreme differences between salinity treatment versus control and ‘Nemaguard’ versus ‘Rootpac 40’. For instance, higher expression of aquaporin *PIP1-2* (*Prupe.5G101400*) was observed in TRR compared to TNR, suggesting that it may be critical for salinity tolerance in *Prunus* (Supplementary Table S3). Aquaporins are known to play positive roles in salinity tolerance in other plant species^45^. A recent report indicates that overexpression of lotus *PIP1-2* (*NnPIP1-2*) in Arabidopsis provides salinity tolerance^46^. NPR4 has been shown to play a negative regulatory role in salinity tolerance^47^. *Prupe.2G080900,* which encodes an ankyrin repeat-containing protein NPR4, was differentially expressed in TNL versus CNL and TRL versus CRL comparisons with a log_2_ fold change value of 6.6 and -6.3, respectively (Supplementary Table S3). These findings propose that upregulation of NPR4 in ‘Nemaguard’ leaf and downregulation in root in response to salinity contributed toward the salt sensitivity of ‘Nemaguard’ compared to ‘Rootpac 40’. F-box protein has been shown to act as a negative regulator in response to salinity tolerance^48^. *Prupe.1G273900* encodes for an F-box protein showed higher expression (log_2_ fold change value of 12.6) in TNR compared to TRR, indicating that upregulation of *Prupe.1G273900* may have contributed negatively toward salinity tolerance in ‘Nemaguard’. Berberine bridge enzyme, betaglucosidase, and MLP like protein are known to contribute to salinity tolerance^49–51^. *Prupe.4G097000*, *Prupe.6G136700,* and *Prupe.1G327700*, which encode berberine bridge enzyme, betaglucosidase, and MLP like protein, respectively, were downregulated in response to salinity in ‘Nemaguard’ leaves compared to control (TNL versus CNL) with log_2_ fold change values of − 9.2, − 8.6, and − 8.3. These observations suggest that downregulation of these three genes under salinity compared to control negatively impacted salinity tolerance in ‘Nemaguard’ (Supplementary Table S3). In TRL versus CRL comparison, *Prupe.1G032800* (log_2_ fold change value of 14.6), which encodes berberine bridge enzyme, was upregulated under salinity compared to control, suggesting that this gene positively contributes to salinity tolerance in ‘Rootpac 40’. GDSL esterase/lipase LTL1 is a positive regulator of salinity tolerance in Arabidopsis^52^. In response to salinity, LTL1 encoded by *Prupe.6G354100* was downregulated both in ‘Nemaguard’ leaves (log_2_ fold change value of -33) and roots (log_2_ fold change value of -27) compared to ‘Rootpac 40’ (TNL versus TRL & TNR versus TRR comparisons). Additionally, under control conditions, lower expression of this gene was observed in ‘Nemaguard’ leaves compared to ‘Rootpac 40’ leaves (CNL versus CRL) (log_2_ fold change value of -26). These facts indicate that downregulation of *Prupe.6G354100* in ‘Nemaguard’ compared to ‘Rootpac 40’ negatively affected salinity tolerance in ‘Nemaguard’. Altogether, differential gene expression analysis revealed that downregulation of genes that are positive regulators and upregulation of genes that are negative regulators of salinity tolerance contributed to the lower salinity tolerance of ‘Nemaguard’, and the opposite expressions contributed to the higher salinity tolerance in ‘Rootpac 40’.

## Conclusion

Salinity tolerance is a complex trait, which is controlled by multiple pathways and many associated genes. Multiple parameters were examined to evaluate the performance of two almond rootstocks, ‘Rootpac 40’ and ‘Nemaguard’, in response to salinity stress. Our findings established that ‘Rootpac 40’ is salt-tolerant- and ‘Nemaguard’ is salt-sensitive. From the biological aspect, the least change in leaf proline concentration, accumulation of leaf Na and Cl, and the higher accumulation of leaf K under salinity were reliable indicators of salinity tolerance and were found in ‘Rootpac 40’. Our transcriptome studies of these two genotypes in response to salinity revealed several genes and pathways critical for salinity tolerance in *Prunus*. We also found that higher expression of CBL10, AKT1, KUP, and chloride channels matched the status of tissue accumulation of Na, K, and Cl, in ‘Rootpac 40’ compared to ‘Nemaguard’. A range of DEGs identified in this study can be used as putative candidates for verification of their biological roles in response to salinity by employing the reverse genetics approaches. Functionally validated genes will be immensely valuable to geneticists and almond breeders for developing highly salt-tolerant rootstocks, essential for successful almond cultivation under saline conditions.

## Materials and methods

### Plant material and salt treatment

All experiments were performed at the United States Salinity Laboratory (USDA-ARS) in Riverside, CA. ‘Nemaguard’ and ‘Rootpac 40’ plants were obtained from Burchell and Agromillora nurseries, respectively, and transplanted into 6 L pots containing 1:1 sandy loam soil: sand. The experiment was conducted in a randomized complete block design with both rootstocks, three plants per replication (each pot containing one plant), three replications for control and saline water treatment. The compositions of both control and saline treatments are indicated in Supplementary Table S12. Riverside city water was used as control, and saline water composition was selected based on our previous study conducted with almond rootstocks^21^. The concentrations of NPK nutrients were the same for control and treatment water. The electrical conductivity (EC_w_) of control irrigation was 1.36 dS m^−1^, and the EC_w_ for treatment saline irrigation was 3.0 dS m^−1^. The pH of both control and treatments was maintained between 7.3 and 7.6. Every other day, 600 ml of control or saline water was applied to each plant. All plants were treated for 10 months.

To study gene expression at the transcriptome level, approximately 1-year-old ‘Nemaguard’ and ‘Rootpac 40’ rootstocks were irrigated with Riverside city water for control and high saline water dominant in sodium and chloride (Supplementary Table S12). Subsequently, 48 h after salinity treatment, roots and leaves were collected from three biological replicates (one plant/biological replicate) for RNA isolation.

### Trunk diameter measurement, survival rate, and leaf mineral element analyses

Before the beginning of the experiment, a vernier caliper was used to measure the trunk diameter of all plants 10 cm above the soil level. At the end of treatment, final trunk diameter data were collected to calculate relative change. After completion of treatment, the survival rate data of ‘Nemaguard’ and ‘Rootpac 40’ were collected. Leaf samples were collected for mineral element analysis eight weeks after the initiation of the treatments. Tissue samples were dried in an oven. Subsequently, a Milestone Ethos E.Z. microwave was used for the digestion of the dried leaf samples. Macro- and micronutrient mineral elements were analyzed using Perkin Elmer Optima ICP OES. Labconco chloridometer was used for chloride content analysis.

### Physiological and biochemical analysis

Measurement of stomatal conductance and photosynthetic parameters were performed using Li-Cor 6400 Photosynthesis System (Li-Cor Biosciences, Lincoln, NE, USA) after eight weeks from the starting day of salt treatment. The details about the measurement method have been described in a previous report^21^. Leaf harvest and measurement and analyses of proline, total phenolics, and hydrophilic antioxidant capacity were performed as described previously^21^.

### RNA-Seq experimental design, RNA extraction, and transcript sequencing

To understand the salinity tolerance mechanism at the transcriptome level of salt-tolerant ‘Rootpac 40’ and salt-sensitive ‘Nemaguard’ rootstocks, we performed a three-factor RNA-Seq experiment using two levels per factor to identify differential gene expression between the following variables: (1) treatment type (control versus salt treatment); (2) rootstock (salt-sensitive, ‘Nemaguard’ versus salt-tolerant, ‘Rootpac 40’); and (3) tissue type (leaf versus root). TRIzol reagent (Invitrogen, Carlsbad, CA, USA) was used for total RNA isolation from leaf and root tissues. Subsequently, all RNA samples were treated with DNase I to remove any DNA contamination (Thermo Scientific, Waltham, MA, USA). The quality and quantity of all RNA samples were checked using Bioanalyzer (Agilent Technologies, USA) and Nanophotometer (IMPLEN, CA, USA). Purification of mRNA from total RNA was performed using Poly-T oligo-attached magnetic beads. NEBNext Ultra RNA library prep kit for Illumina (NEB) was used for construction sequencing libraries. Illumina HiSeq platform was employed for RNA sequencing and to generate 150 bp paired-end reads (Novogene Corp. Inc., Sacramento, CA). To obtain clean sequences, raw reads were subsequently trimmed and clipped of adaptors using Trimmomatic^53^. Clean reads were analyzed for quality scores, Q20 (error 1 in 100) and Q30 (error 1 in 1000), and GC contents were analyzed utilizing FASTQC (http://www.bioinformatics.babraham.ac.uk/projects/fastqc/). Reads deemed to be clean were then used for further analysis.

### Transcriptome assembly mapping

Raw reads were stored in a left.fq.gz file and a right.fq.gz file. A shell script was written to quantify the pair-end reads using the mapping-based mode on Salmon^54^. The index was created using the *Prunus persica* NCBIv2.1 Transcriptome assembly^55^. The “validatemappings” flag was set so that the more sensitive selective-alignment algorithm could be used^55^. The “gcBias” flag was also set to allow Salmon to correct for potential fragment-level guanine-cytosine content biases in the input data. Quantification files containing the read counts were then output with the .sf extension.

### Differential gene expression analysis

The biomaRt package^56,57^ (2.42.0) was used to create a table of Ensembl gene ids for *P. persica*. Using the tximport package (1.14.0)^58^, .sf files from Salmon were then imported and mapped to their corresponding Ensembl gene name. A matrix was created containing the sample name as the column, gene ID as the row, and the corresponding read counts as the observations. Genes with lower than 5 counts were filtered out of the data, as they would not be of much use in the analysis. Differential expression analysis was performed using DESeq2 (1.26.0)^59^. The package uses a model based on the negative binomial distribution to estimate variance-mean dependence of the gene count data^59^. Adjusted* p*-values (*q*-values) were generated using Benjamini and Hochberg’s method to control the false discovery rate^59^. The significance cutoff was set to α = 0.05. Genes with an adjusted *p*-value lower than 0.05 and a log_2_ fold change with an absolute value greater than 2 were deemed differentially expressed. If a gene satisfied both *q*-value ≤ 0.05 and |log_2_(fold change)|≥ 2, it was considered significantly differentially expressed.

Using the DEGs, Venn diagrams and heatmaps were constructed. The Venn diagrams were made using the “VennDiagram” package in R^60^. Heatmaps were generated using the “pheatmap” package in R (https://cran.r-project.org/web/packages/pheatmap/index.html). For clustering purposes, a Z-score transformation ($Z=\frac{x- \mu }{\sigma }$) was performed on the matrix of pooled read counts for differentially expressed genes.

### Gene ontology annotation and enrichment analysis of DEGs

Gene ontology (GO) analysis of the differentially expressed genes was done using the GOseq (1.38.0) package in R^61^. We assumed that, within a category, all genes have the same probability of being chosen. This allowed us to use the Wallenius non-central hypergeometric distribution to perform the GO enrichment test. First, an over-representation test was performed using the DEGs as the input set. Next, the over-representation of the DEGs was tested for using an adjusted *p*-value cutoff of α = 0.05. The effect size calculation was then performed by taking the p-value and performing a negative 10-base log transformation.

### KEGG enrichment analysis of DEGs

KEGG (Kyoto Encyclopedia of Genes and Genomes, http://www.kegg.jp/) consists of databases containing information regarding high-level functions and utilities in biological systems. KEGG pathway enrichment analysis was done using the ClusterProfiler (3.18.1) in R^62^. To perform the enrichment analysis, DEGs were converted from their ensemble id to their Entrez id equivalent. KEGG pathways in the DEGs were then compared to pathways associated with the entire genome background using the hypergeometric distribution and adjusted for a high FDR. KEGG terms with an adjusted *p*-value less than 0.05 were deemed to be significantly enriched. The effect size calculation was then performed by taking the *p*-value and performing a negative 10-base log transformation.

### Functional analysis and visualization

Keywords for specified paths were searched for in each comparison using the built-in “grep” R function. Genes containing specific keywords were colored depending on the value of their log_2_ fold change within the comparison. For example, genes with a log_2_ fold change greater than 0 were colored blue for upregulation, and genes with a log_2_ fold change less than 0 were colored red for downregulation.

### Transporter analysis

Tables containing Transporter families were obtained from the Transporter Classification Database (https://tcdb.org/). Pfam IDs were used to map the genes to their associated families and superfamilies.

### Quantitative reverse transcription PCR (qRT-PCR)

Forty-one genes were selected randomly for qRT-PCR analysis to validate RNA-Seq data (Supplementary Tables S4 & S5). qRT-PCR assays were performed as described previously^32^. RNA samples that were used for RNA-Seq library preparation were also used for qRT-PCR analyses. To remove DNA contamination, RNA samples were treated with DNase I (New England Biolabs Inc., Ipswich, MA). The Bio-Rad CFX96 System (Bio-Rad Laboratories, Hercules, CA) was used to perform qRT-PCR using the iTaq Universal SYBR Green One-Step Kit. All qRT-PCR reactions were performed in a total volume of 10 μl containing 20 ng total RNA, 0.75 μM of each of the primers, 5 μl of 2 × one-step SYBR® Green Reaction mix, and 0.125 μl iScript™ Reverse Transcriptase. The qRT-PCR conditions used were: 50 °C for 10 min, 95 °C for 1 min, followed by 40 cycles of 95 °C denaturation for 10 s, 57 °C annealing for 30 s, and 68 °C extension for 30 s. *PpUBQ10*, *PpRPII*, *PpTEF2* were used as the reference for the analyses. All assays were conducted using RNA from three biological replicates and two technical replicates. To check DEGs, the cycle threshold (CT) values of all tested genes were compared to the reference, and subsequently, the difference in expressions was calculated.

The use of plants in the present study complies with international, national, and/or institutional guidelines.

## Supplementary Information

Supplementary Figures.

Supplementary Table S1.

Supplementary Table S2.

Supplementary Table S3.

Supplementary Table S4.

Supplementary Table S5.

Supplementary Table S6.

Supplementary Table S7.

Supplementary Table S8.

Supplementary Table S9.

Supplementary Table S10.

Supplementary Table S11.

Supplementary Table S12.

## Data Availability

Illumina HiSeq generated RNA-Seq reads are available in NCBI Sequence Read Archive (SRA) for the Bioproject: PRJNA732909 (https://www.ncbi.nlm.nih.gov/Traces/study/?acc=%20PRJNA732909&o=acc_s%3Aa). Additional datasets supporting this research are included in the paper and as supplementary materials.

## Abbreviations

qRT-PCR: Quantitative reverse transcription PCR
ORAC: Oxygen radical absorbance capacity
SPAD: Soil Plant Analysis Development
EC: Electrical conductivity
