# Supplementary material for: Morphological, physiological, biochemical, and transcriptome studies reveal the importance of transporters and stress signaling pathways during salinity stress in Prunus
Source: Sci Rep. 2022 Jan 24;12:1274. doi: 10.1038/s41598-022-05202-1 (PMC8786923; doi:10.1038/s41598-022-05202-1)
Supplement: Supplementary file 1 — Supplementary Figures. [file 41598_2022_5202_MOESM1_ESM.pdf]

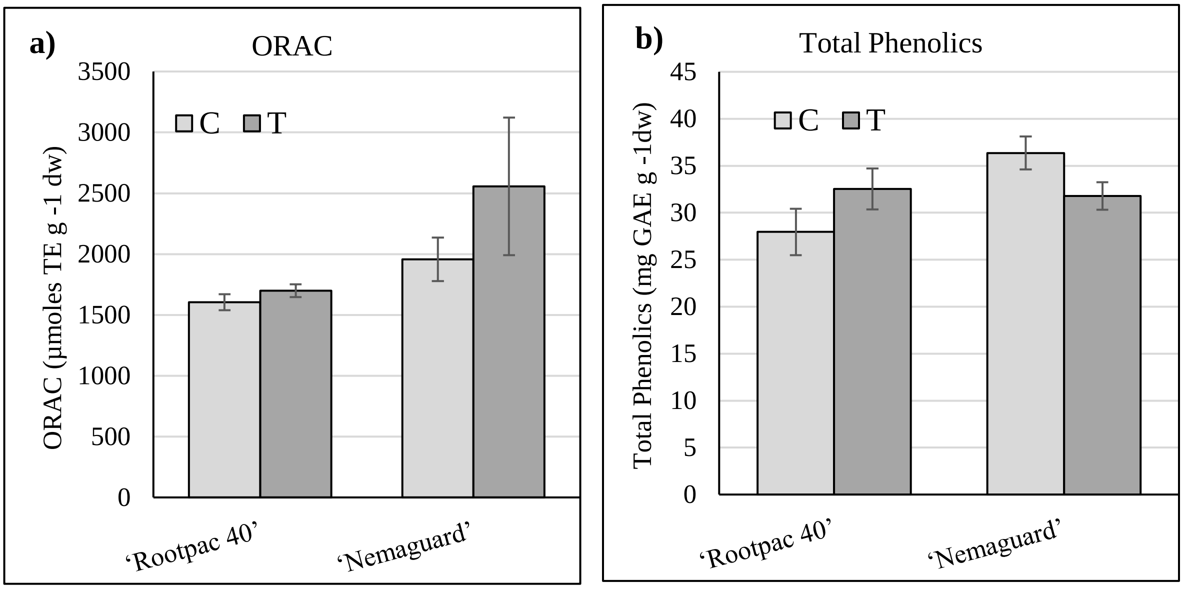

Supplementary Fig. S1. Antioxidant capacity of 'Rootpac 40' and 'Nemaguard' rootstocks irrigated with control and saline water. a) Hydrophilic oxygen radical absorbance capacity (ORAC). ORAC units are shown in the Y-axis in micromoles of trolox equivalents per gram of dry weight (µmoles·TE·g^{-1} dw). b) Total phenolics. The Y-axis indicates milligrams of gallic acid equivalents per gram of dry weight (mg·GAE·g–1 dw). Error bars represent standard errors.

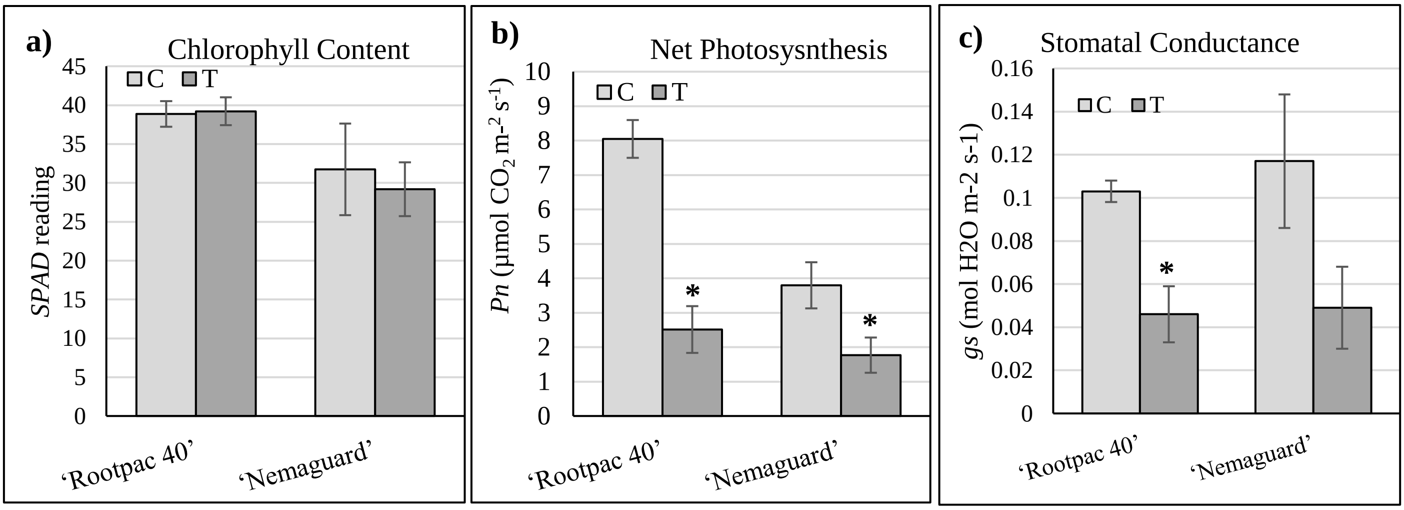

Supplementary Fig. S2. Physiological responses of 'Rootpac 40' and 'Nemaguard' rootstocks under control and saline treatments. a) Chlorophyll content (*Chla* + *Chlb*). b) Net photosynthesis (*Pn*). c) stomatal conductance (*gs*). Error bars represent standard errors. Asterisks show differences (t-test $p \leq 0.05$) between the control (C) and treatment (T) of the indicated rootstock.

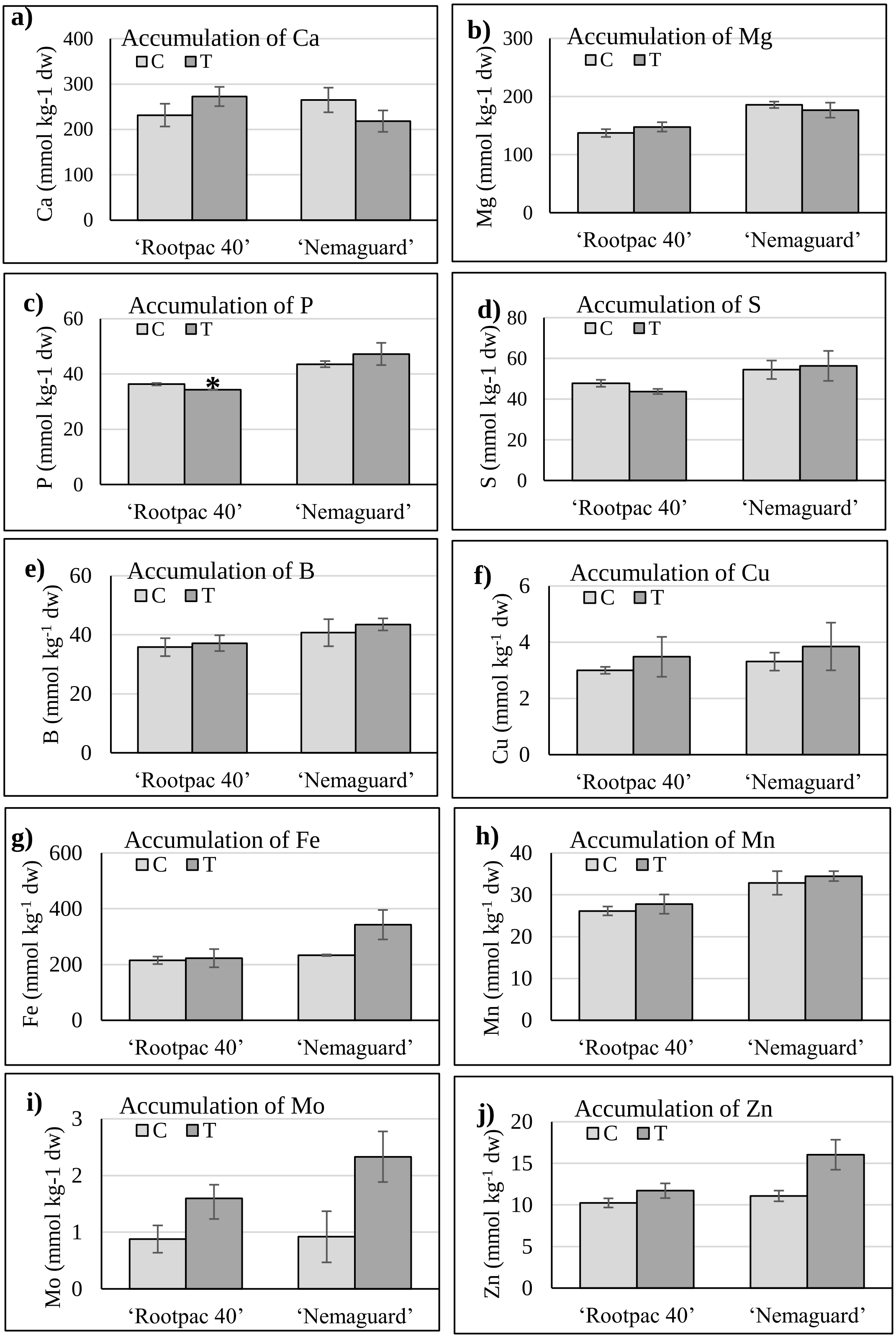

Supplementary Figure S3. Leaf ion concentrations of 'Rootpac 40' and 'Nemaguard' under control and saline treatments). Error bars represent standard errors. Asterisk indicates differences (t-test $p \leq 0.05$) between the control (C) and treatment (T) of the indicated rootstock. a) Calcium (Ca). b) Magnesium (Mg). c) Phosphorus (P). d) Sulphur (S). e) Boron (B). f) Copper (Cu). g) Iron (Fe). h) Manganese (Mn). i) Molybdenum (Mo). j) Zinc (Zn).

Supplemental Figure S4

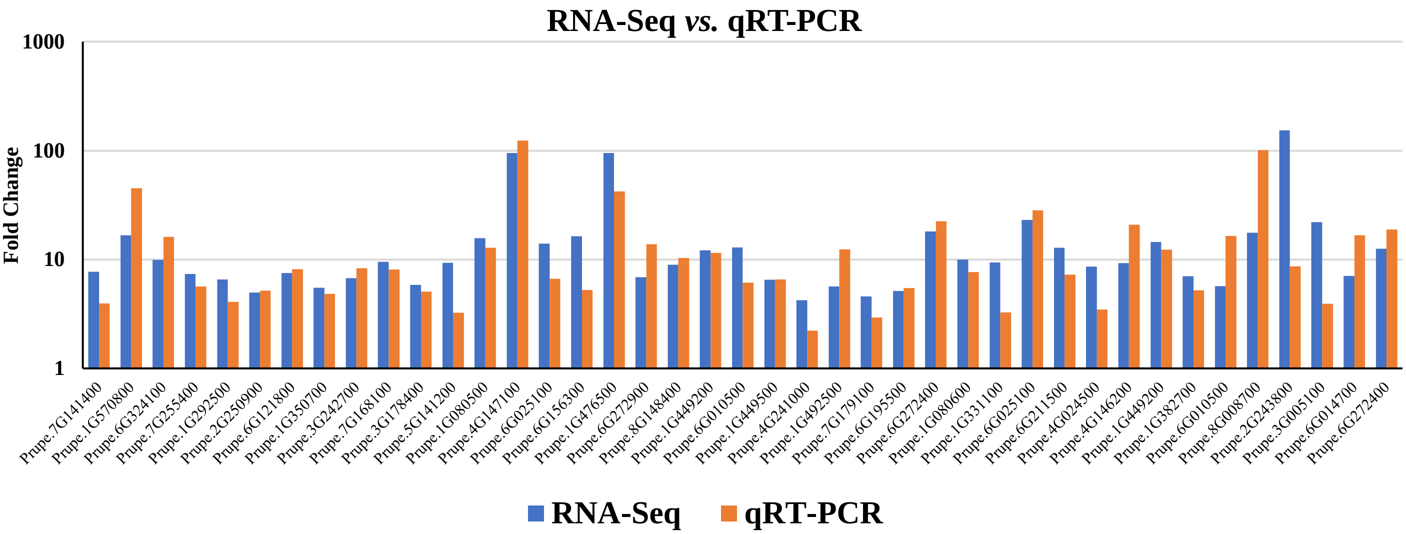

Supplementary Fig. S4. Comparison of fold-change of gene expression between RNA-Seq and qRT-PCR of the indicated DEGs. Genes tested are shown in the X-axis, and the fold-change values of the gene expression values are shown in the Y-axis.
