# Supplementary material for: Morphological, physiological, biochemical, and transcriptome studies reveal the importance of transporters and stress signaling pathways during salinity stress in Prunus
Source: Sci Rep. 2022 Jan 24;12:1274. doi: 10.1038/s41598-022-05202-1 (PMC8786923; doi:10.1038/s41598-022-05202-1)
Supplement: Supplementary file 2 — Supplementary Table S1. [file 41598_2022_5202_MOESM2_ESM.docx]

**Supplementary Table S1**. Experimental setup shows leaf and root samples from two rootstocks ‘Nemaguard’ (salt-sensitive) and ‘Rootpac 40’ (salt-tolerant) subjected to control and salt treatment

| **Tissue** | **Replicates** | **Control** | | **Salt treatment** | |
| --- | --- | --- | --- | --- | --- |
|  |  | **‘Nemaguard’** | **‘Rootpac 40’** | **‘Nemaguard’** | **‘Rootpac 40’** |
| **Leaf** | R1 | CNL-1 | CRL-1 | TNL-1 | TRL-1 |
|  | R2 | CNL-2 | CRL-2 | TNL-2 | TRL-2 |
|  | R3 | CNL-3 | CRL-3 | TNL-3 | TRL-3 |
| **Root** | R1 | CNR-1 | CRR-1 | TNR-1 | TRR-1 |
|  | R2 | CNR-2 | CRR-2 | TNR-2 | TRR-2 |
|  | R3 | CNR-3 | CRR-3 | TNR-3 | TRR-3 |
