# Supplementary material for: Morphological, physiological, biochemical, and transcriptome studies reveal the importance of transporters and stress signaling pathways during salinity stress in Prunus
Source: Sci Rep. 2022 Jan 24;12:1274. doi: 10.1038/s41598-022-05202-1 (PMC8786923; doi:10.1038/s41598-022-05202-1)
Supplement: Supplementary file 13 — Supplementary Table S12. [file 41598_2022_5202_MOESM13_ESM.docx]

**Supplementary Table S12. Control and treatment irrigation water composition**

| **Treatment** | **Salt composition** |
| --- | --- |
| Control (C) | Non-saline control [Na^+^ 1.65 mmol_c_ L^−1^, K^+^ 6.5 mmol_c_ L^−1^, PO4^3−^ 1.5 mmol_c_ L^−1^, Mg^2+^ 1.3 mmol_c_ L^−1^, SO_4_^2−^ 1.5 mmol_c_ L^−1^, Cl^−^ 1.5 mmol_c_ L^−1^, NO_3_^−^ 5 mmol_c_ L^−1^ and micronutrients] |
| Salinity Treatment (T) | Mixed cations (Ca^2+^ = 1.25 Mg^2+^ = 0.25 Na^+^) with predominantly chloride (SO_4_^2−^ = 0.2 Cl^−^) [Na^+^ 15.5 mmol_c_ L^−1^, Ca^2+^ 3.8 mmol_c_ L^−1^, K^+^ 6.5 mmol_c_L^−1^, PO_4_^3−^ 1.5 mmol_c_ L^−1^, Mg^2+^ 3.1 mmol_c_ L^−1^, SO_4_^2−^ 3.8 mmol_c_ L^−1^, Cl^−^ 19 mmol_c_ L^−1^, NO^3−^ 5 mmol_c_ L^−1^ and micronutrients] |
